# Genome-Wide Association Study of Reproductive Traits in Large White Pigs

**DOI:** 10.3390/ani14192874

**Published:** 2024-10-06

**Authors:** Yifeng Hong, Cheng Tan, Xiaoyan He, Dan Wu, Yuxing Zhang, Changxu Song, Zhenfang Wu

**Affiliations:** 1 College of Animal Science and National Engineering Research Center for Breeding Swine Industry, South China Agricultural University, Guangzhou 510642, China; hongyf0504@163.com; 2 State Key Laboratory of Swine and Poultry Breeding Industry, Guangzhou 510640, China; tancheng200508@163.com; 3 Yunfu Branch, Guangdong Laboratory for Lingnan Modern Agriculture, Yunfu 527300, China; elainecn@163.com (X.H.); 15013019352@163.com (D.W.); zhangyuxing1998@163.com (Y.Z.); 4 National Engineering Research Center for Breeding Swine Industry, WENS Foodstuff Group Co., Ltd., Yunfu 527400, China

**Keywords:** Large White pigs, litter traits, GWAS, SNPs, candidate genes

## Abstract

**Simple Summary:**

Understanding the genetics behind the reproductive traits of pigs is crucial for enhancing productivity and economic outcomes in the pork industry. In this study, we investigated the genetic factors influencing reproductive characteristics in Large White pigs. By analyzing the genetic data of 2237 sows from southern China, we identified specific genes and genetic markers associated with important reproductive traits, such as litter size. Our findings provide valuable insights that could help in developing targeted breeding strategies aimed at improving these traits. This research is not only significant for farmers and breeders but also contributes to a more sustainable and efficient approach to pig farming.

**Abstract:**

(1) Background: Reproductive performance is crucial for the pork industry’s success. The Large White pig is central to this, yet the genetic factors influencing its reproductive traits are not well understood, highlighting the need for further research. (2) Methods: This study utilized Genome-Wide Association Studies to explore the genetic basis of reproductive traits in the Large White pig. We collected data from 2237 Large White sows across four breeding herds in southern China, focusing on eight reproductive traits. Statistical analyses included principal component analysis, linkage disequilibrium analysis, and univariate linear mixed models to identify significant single-nucleotide polymorphisms and candidate genes. (3) Results: Forty-five significantly related SNPs and 17 potential candidate genes associated with litter traits were identified. Individuals with the TT genotype at SNP rs341909772 showed an increase of 1.24 in the number of piglets born alive (NBA) and 1.25 in the number of healthy births (NHBs) compared with those with the CC genotype. (4) Conclusions: The SNPs and genes identified in this study offer insights into the genetics of reproductive traits in the Large White pig, potentially guiding the development of breeding strategies to improve litter size.

## 1. Introduction

Reproductive performance is a critical determinant of productivity and economic viability in the pork industry. Among various breeds, the Large White pig is renowned for its adaptability and reproductive performance, making it a staple in commercial pig farming globally. However, despite the breed’s importance, genetic factors underlying its reproductive traits remain incompletely understood [1]. The complexity of reproductive traits, which are influenced by a myriad of genetic and environmental factors, poses significant challenges in enhancing trait predictability and selection efficiency [2]. Particularly, litter size is a vital trait in the pork industry, directly impacting the economic outcomes of pig farming.

Reproductive traits in pigs, such as litter size, fertility, and piglet survival, are quintessential quantitative traits with low to moderate heritability [3]. This genetic architecture suggests that these traits are controlled by numerous genes with small effects rather than by a few major genes. Traditional phenotypic selection methods have achieved limited success, primarily due to the low heritability of these traits, which leads to slow genetic progress. Consequently, there is a pressing need for more precise and effective genetic improvement strategies.

In recent years, Genome-Wide Association Studies (GWASs) have emerged as a powerful tool for dissecting the genetic basis of complex traits in livestock. GWAS allows for the examination of the entire genome to identify associations between genetic markers and traits of interest. This approach has successfully identified numerous single-nucleotide polymorphisms (SNPs) and candidate genes associated with economically important traits in pigs, including coat color [4], meat quality [5], and reproductive performance [6]. Notably, GWAS on reproductive traits in pigs has revealed several loci and genes potentially affecting litter size and piglet viability, which are crucial for improving reproductive efficiency.

The present study aimed to conduct a comprehensive GWAS to identify SNPs and candidate genes associated with eight reproductive traits related to litter size in Large White pigs. The findings from this study are expected to contribute to the development of molecular breeding strategies that could expedite genetic improvements and, thus, enhance the productivity and profitability of the pork industry.

## 2. Materials and Methods

### 2.1. Animals and Traits

The animals used in this study were sourced from a swine breeding herd located in southern China, owned and managed by Wens Foodstuff Group Co., Ltd., based in Yunfu China. Data collection from these herds took place between November 2021 and May 2024. A total of 3368 records (total number born, TNB), only including first and second litters, were observed from 2237 Large White sows. The reproductive traits assessed included TNB, NBA, NHB, the rate of healthy births (rNHBs), the number of weak births (NWBs), the number of deformed fetuses (NDFs), the number of stillborn fetuses (NSBs), and the number of mummified pigs (MUMM). TNB represents the total number of piglets born in a single birthing event and is calculated as the sum of NBA, NSB, and MUMM. NBA is the total count of all surviving piglets, comprising NHB, NWB, and NDF. NHB denotes piglets with a birth weight of no less than 1.0 kg, whereas NWB refers to those piglets weighing less than 1.0 kg; however, neither NSB nor NWB exhibits genetic defects. The rNHB ratio is defined as the proportion of NHB to TNB. NDF pertains to piglets born with genetic anomalies, such as limb malformations or reproductive system disorders. NSB indicates the count of piglets that were stillborn or died during gestation. MUMM refers to cases where a pig fetus dies or undergoes degeneration after the 35th day of gestation without leading to a miscarriage. Ultimately, the eight traits, TNB, NBA, NHB, rNHB, NWB, NDF, NSB, and MUMM—were utilized for further analysis, with details provided in Table 1.

### 2.2. Genotypes and Quality Control

A total of 2237 tail tissue samples from Large White sows were collected, and genomic DNA was extracted using the QIAamp DNA Mini Kit (QIAGEN, Shanghai, China). The DNA quality was subsequently assessed via spectrophotometry NanoDropTM 2000 (Thermo Fisher Scientific, Waltham, MA, USA) and gel electrophoresis. Samples meeting the quality standards (minimum concentration of 50 ng/μL and an A260/A280 ratio between 1.8 and 2.0) were genotyped using the Affymetrix Wens 55K SNP Chip. Quality control measures for the genotyping data were implemented using Plink 1.90 [7], setting criteria such as a Hardy-Weinberg equilibrium threshold of 10^−6^, a minor allele frequency of 0.01, a minimum individual call rate of 0.95, and an SNP call rate of 0.99. Ultimately, 39,580 SNP markers met the quality control standards across all 2237 Large White pigs.

### 2.3. Statistical Analysis

Principal component analysis (PCA) was conducted using GCTA software version 1.93.2 beta [8], while linkage disequilibrium (LD) analysis was performed with PopLDdency software version 3.40 [9]. We analyzed the average LD decay distance across the whole genome where r2 = 0.2. Heritability was estimated using the GCTA software version 1.93.2 beta. The GWAS employed univariate linear mixed models (MLMs) via the GEMMA software version 0.98.5 [10], modeled as Y=Zβ+Wa+u+e, where Y represents the vector of phenotypic records, β is the vector of the corresponding coefficients including the intercept, a is the effect size of the marker, u is the vector of random effects, e is the vector of errors, Z is a matrix of covariates (fixed effect including farm-year-moth), and W is the vector of marker genotypes. The random effect u and errors e are assumed to follow multivariate normal distributions: ($u\;∼\;{MVN}_{n}\;\left(0,\lambda \tau^{-1}K\right),\;e\;∼\;{MVN}_{n}\;(0,\tau^{-1}I_{n})$), where λ is the ratio between the two variance components; K is a known relatedness matrix; $I_{n}$ is the identity matrix; and ${MVN}_{n}$ denotes the dimensional multivariate normal distribution. To establish thresholds for the genome-wide significance and suggestive significance, we based our calculation on 14,443 effectively independent SNP derived from independent markers and an LD block (defined as a set of SNPs with pairwise r2 values greater than 0.80). Consequently, the genome-wide significance threshold was set at 0.05/14,443 = 3.46 × 10^−6^, and the genome-wide suggestive significance threshold was set at 1/14,443 = 6.92 × 10^−5^. The GWAS results were visualized using the CMplot package in R version 4.3.2, generating Manhattan and quantile–quantile (Q-Q) plots. GWAS frequently identifies clusters of significant SNPs associated with a target trait within a putative region, likely due to the LD among them. To delineate the independence of all significant signals within such a region, conditional analyses were conducted using a mixed linear model (MLM), where the genotypes of peak SNPs were incorporated as covariates [11].

### 2.4. Identification of Candidate Genes and Analysis of Functional Enrichment

The physical locations of all SNPs and functional genes are mapped to the latest Sus scrofa 11.1 genome assembly, accessible at [https://ensembl.org/Sus_scrofa/Info/Index (accessed on 15 July 2024)]. Annotation was performed by identifying the nearest functional gene to each significant SNP, along with potential candidate genes selected based on their physiological and biochemical functions. Subsequent Gene Ontology (GO) term annotation and Kyoto Encyclopedia of Genes and Genomes (KEGG) pathway enrichment analysis were conducted using KOBAS 3.0 [12]. Biological process GO terms and KEGG pathways were then selected based on an adjusted *p*-value threshold of <0.01.

## 3. Results and Discussion

Over recent decades, the selective breeding of Large White pigs has primarily concentrated on enhancing traits such as litter size to augment production efficiency and economic viability within the swine industry. Although this strategy has effectively increased yields, it has inadvertently escalated a range of reproductive challenges. These include NWB, NDF, NSB, and MUMM, thereby highlighting the intricate nature of reproductive traits governed by a complex interplay of genetic and environmental factors. The GWAS on reproductive traits in Large White pigs offers an exhaustive analysis aimed at elucidating the genetic underpinnings that influence these reproductive efficiencies and anomalies. By pinpointing specific single SNPs associated with both beneficial and detrimental reproductive outcomes, this research endeavors to establish a foundation for more holistic breeding programs. Such programs aim not only to enhance productivity but also to significantly improve the overall health and welfare of the animals, ensuring sustainable practices within the breeding sector.

### 3.1. Genetic Architecture and LD Decay

PCA depicted in Figure S1 reveals a homogenous genetic background among the pigs studied, indicating an absence of population stratification. This uniformity is crucial in GWAS as it suggests that any association detected between SNP markers and traits is less likely to be confounded by population structure. Further, LD decay analysis, illustrated in Figure S2, shows that LD decays to a correlation coefficient of 0.20 at about 800 kb. This LD decay distance is significant for GWAS as it informs the resolution at which genetic associations can be mapped. A shorter LD decay implies a higher resolution for locating genes associated with traits, whereas a longer LD decay suggests broader genomic regions harboring potential causative variants. In this case, the 800 kb LD decay provides a moderate resolution, facilitating the identification of candidate regions but requiring denser SNP panels or follow-up studies for precise gene mapping.

### 3.2. Heritability

In our study, heritability estimates for reproductive traits such as TNB, NBA, NHB, rNHB, NWB, NDF, NSB, and MUMM in both first and second litters ranged from 0.01 to 0.07, as detailed in Table 1. These low heritability values indicate that genetic factors make only a minimal contribution to the observed variance in these traits among pigs. Consequently, the limited genetic influence reflected by these heritabilities may restrict the effectiveness of GWAS in identifying significant genetic markers associated with these traits. This suggests that although genetic factors do influence these reproductive traits, their overall impact is modest. Many reports have also found these traits to have low heritability, generally ranging from 0.01 to 0.15 [13,14,15]. Therefore, larger sample sizes may be necessary to detect any substantial genetic contributions. Despite these challenges, we have still managed to identify some valuable GWAS findings. Moving forward, increasing the sample size to enhance the statistical power of GWAS is crucial for identifying GWAS findings related to litter size traits.

### 3.3. Genome’Wide Association Studies

**Figure 1:**
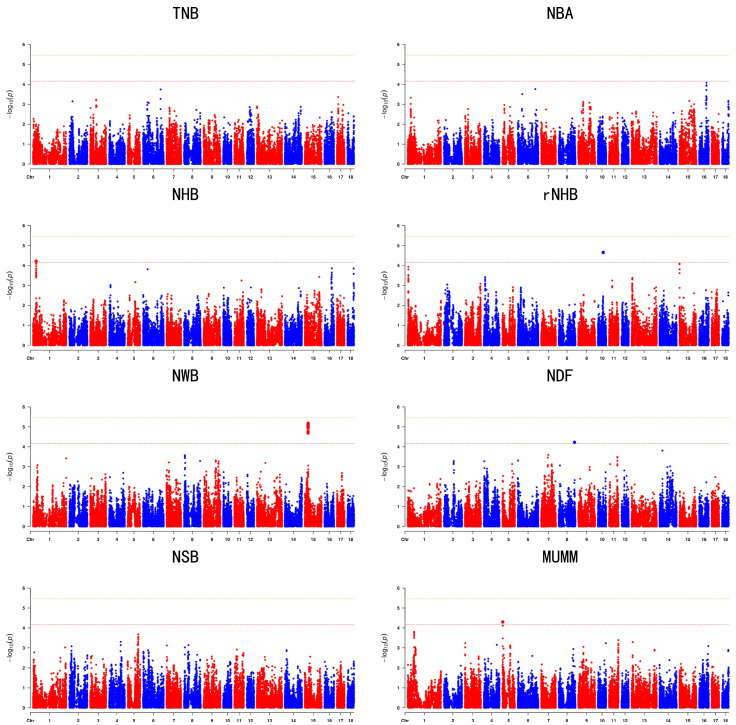
Manhattan plots of GWAS with SNPs for litter traits at the first parity in the Large White populations. In the Manhattan plots, the yellow and red lines represent the 5% genome-wide and chromosome-wide (suggestive) Bonferroni-corrected thresholds, respectively.

**Figure 2:**
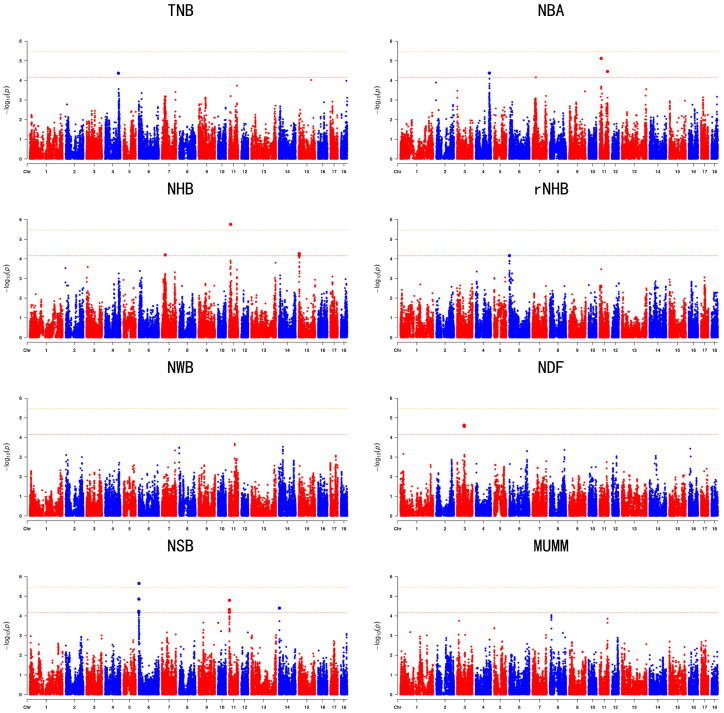
Manhattan plots of GWAS with SNPs for litter traits at the second parity in the Large White populations. In the Manhattan plots, the yellow and red lines represent the 5% genome-wide and chromosome-wide (suggestive) Bonferroni-corrected thresholds, respectively.

The GWAS results are presented in Table 2 and Table 3 and Figure 1 and Figure 2. Specifically, Table 2 and Figure 1 display the GWAS results for the first-parity litter size trait, whereas Table 3 and Figure 2 focus on the GWAS results for the second-parity litter size trait. In total, 45 SNPs were identified across the traits TNB, NBA, NHB, rNHB, NWB, NDF, NSB, and MUMM, including two significant and 43 suggestive SNPs. Specifically, the GWAS results for the first parity identified 21 suggestive SNPs, while the results for the second parity revealed two significant and 23 suggestive SNPs. Q-Q plots were employed to demonstrate the potential inflation of *p*-values, as depicted in Figures S3 and S4. The average genomic inflation factors (λ) for litter traits ranged from 0.98 to 1.02, further indicating minimal population stratification.

The GWAS results for the first and second parity litter size traits reveal distinct genetic influences, as evidenced by the lack of shared quantitative trait loci (QTLs) between them. This disparity could be attributed to several factors. Firstly, genetic variation specific to each parity may be driven by different biological processes, such as maternal age, physiological changes, or environmental adaptations that occur between the first and second pregnancies [16]. Secondly, the reproductive performance in pigs, like many other traits, can be influenced by epigenetic modifications that differ from one parity to another. These modifications can affect gene expression without altering the DNA sequence, potentially leading to different genetic markers being identified in the GWAS of each parity [17]. Additionally, the interaction between genetic and environmental factors can vary across parities, further contributing to the unique genetic landscapes observed [18]. Lastly, the statistical power and the methodology used in identifying significant and suggestive SNPs might also differ slightly, affecting the detection of common QTLs between the two parities. This analysis underscores the complexity of genetic architecture in litter size traits and highlights the need for tailored strategies in genetic selection and management practices for different parities.

### 3.4. Candidate Genes for Litter Traits

An SNP, rs55618047, was significantly associated with TNB traits in the second parity, with the candidate gene PRMT6 identified in close proximity to this SNP. Research indicates that the PRMT6 gene may be associated with birth weight traits in pigs. A previous genome-wide association study (GWAS) identified the *PRMT6* gene variant rs12097821, located at 1p13.3, as a genetic susceptibility locus for nonobstructive azoospermia (NOA) in the Han Chinese population [19]. Similarly, in a GWAS on Polish Holstein-Friesian bulls, *PRMT6* was highlighted due to its proximity to significant SNPs—specifically rs109154964 and rs108965556 on chromosome 3—which are associated with variations in sperm concentration [20]. This gene has also been identified as a promising candidate for further research into the progression of geographic atrophy, a condition urgently requiring effective treatments [21]. Additionally, the *PRMT6* variant rs2232016 has been recognized as a new susceptibility locus for gestational diabetes mellitus (GDM) in the Chinese Han population. In a case-control study involving North Macedonian men with idiopathic infertility, the rs12097821 variant in the *PRMT6* gene was analyzed [22]. Although the study revealed no significant differences in genotype distributions between the infertile men and controls, a subsequent meta-analysis confirmed the association of rs12097821 with the risk of idiopathic male infertility [23]. These findings underscore the diverse roles of *PRMT6* in reproductive traits and diseases, highlighting its importance in genetic research and potential therapeutic targets.

**Figure 3:**
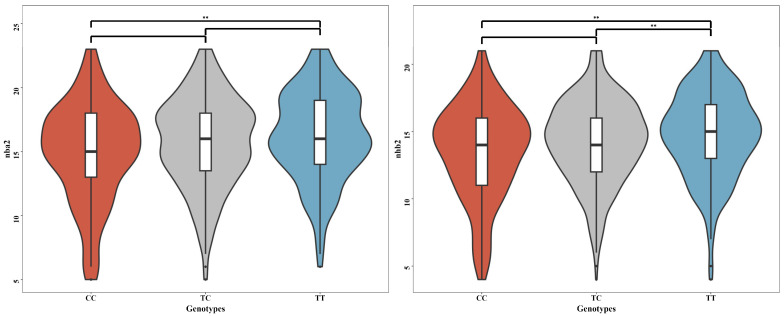
The genotype effect plot of the pleiotropic SNP rs341909772, which is associated with both NBA and NHB in the Large White population at the second parity, is displayed. On the left, the plot shows the effect of SNP rs341909772 on NBA, and on the right, the effect on NHB (** *p* < 0.001).

The SNP rs341909772 was significantly associated with both NBA traits in the second parity and NHB traits in the second parity, with the candidate gene *COG6* identified near this SNP. Although there is no existing research linking the *COG6* gene to reproductive or litter traits in pigs, it has been implicated in human health conditions. Specifically, the *COG6* gene (rs7993214) was identified as a susceptibility locus for juvenile idiopathic arthritis in a study, which found an odds ratio (OR) of 0.76 and a *p*-value of (1.10 × 10^−5^), suggesting it may have a protective effect against the disease [24]. Additionally, while this gene was investigated as a potential genetic risk factor for psoriasis in the Chinese population, it did not demonstrate a significant association in this group, contrasting with findings from Western populations reported in other Genome-Wide Association Studies [25]. Furthermore, a GWAS identified a significant bone mineral density (BMD) locus on Chromosome 3, which includes the *COG6* gene within a 300 Kbp region known to influence BMD. This locus coincides with a previously identified quantitative trait locus (QTL) for BMD, underscoring a potential role for *COG6* in the regulation of bone density [26]. Individuals with the TT genotype at SNP rs341909772 demonstrated higher NBA (1.24) and NHB (1.25) values compared with those with the CC genotype and also exhibited higher NBA (0.45) and NHB (0.52) compared with those with the CT genotype (Figure 3). Although this pleiotropic SNP may not be the causal mutation, it has been shown to possess significant molecular breeding value in GWAS.

Two additional SNPs, rs3474737609 and rs81431697, have been significantly linked to NBA traits in the second parity, with the candidate genes *OLFM3* and *DOCK9* located nearby. In a GWAS of Colombian creole cattle breeds, Blanco Orejinegro and Sanmartinero, the *OLFML3* gene emerged as a key player in reproductive traits, notably affecting the age at first calving and calving intervals [27]. Moreover, the proximity of *OLFM3* to a newly identified Parkinson’s disease locus on chromosome 1p21 suggests its potential role in the genetic landscape of sporadic Parkinson’s disease [28]. This association may indicate a specific genetic influence on the development of the disease. The study also underscores the importance of the *DOCK9* gene in the genetic profile of domestic pigs, indicating its influence on key phenotypic traits [29]. Alongside genes such as *ACACA* and *MECR*, *DOCK9* is involved in the complex interaction between genotype and phenotype, particularly in traits relevant to domestication and breeding. Identified within a co-expression module enriched for genes implicated by bone mineral density GWAS, *DOCK9* is highlighted as a potential causal genetic driver of BMD in humans [30]. It is located within the BMD GWAS loci, which has colocalizing eQTLs and exhibits altered BMD in mouse knockouts. Collectively, these findings enhance our understanding of the genetic factors that shape economic reproduction traits in livestock, highlighting the potential for targeted genetic improvements in breeding programs. Individuals possessing the GG genotype at SNP rs81431697 exhibited an average increase of 1.31 in NBA values compared with those with the AA genotype and a 0.60 increase compared with individuals with the GA genotype (Figure S5). Additionally, this SNP also possesses significant breeding value.

Moreover, SNP (rs55618047) was significantly associated with NHB traits in the first parity, with the candidate gene *TXLNB* located near this SNP. Three SNPs (rs341878379, rs1108768276, rs320524315) demonstrated significant associations with NHB traits in the second parity, with the candidate genes *COL21A1* and *ENSSSCG00000053606* identified near these SNPs. Furthermore, one SNP (rs81294332) showed a significant association with rNHB traits in the first parity, with the candidate gene *SLC39A12* located in close proximity. Another SNP (rs320041489) was significantly associated with rNHB traits in the second parity, with the candidate gene *MTHFSD* identified near this SNP. *TXLNB*, which encodes a coiled-coil domain protein, has been identified as a critical regulator of cardiac proteostasis. It is capable of modulating protein synthesis, degradation, and autophagic processes in response to cardiac stress. Overexpression of *TXLNB* in neonatal rat cardiomyocytes modifies the typical hypertrophic response to phenylephrine by inhibiting the increase in cell size, highlighting its essential role in maintaining protein homeostasis during cardiac hypertrophy [31]. Research has indicated that specific causative mutations in the *COL21A1* gene may play a pivotal role in the regulation of growth and carcass traits in pigs. These mutations in *COL21A1* are potentially linked to critical biological processes that influence meat quality and yield [32]. GWAS have identified the *SLC39A12* gene as significantly associated with sperm abnormalities, such as damaged cell necks and tails, in cryopreserved Holstein bull semen, suggesting its vital role in preserving the structural integrity of sperm during the cryopreservation process [33]. Additionally, real-time quantitative PCR was employed to validate a 496 kb copy number variation (CNV) region containing the *MTHFSD* gene on chromosome 6 in Xiang pigs, revealing significant copy number variations associated with reproductive traits. The presence of CNVs in the *MTHFSD* gene, which functions as an RNA-binding protein, has been shown to significantly affect litter size in Xiang pigs, indicating its crucial role in regulating mRNA metabolism and pig reproduction [34]. Further research has identified an SNP (rs336610321) in *MTHFSD* that is significantly associated with TNB in pigs [35]. Individuals carrying the GG genotype at SNP rs81294332 demonstrated an average rNHB value increase of 6.29 compared with those with the AA genotype and a 3.44 increase relative to carriers of the GA genotype (Figure S6). Therefore, this SNP also holds significant breeding value.

**Figure 4:**
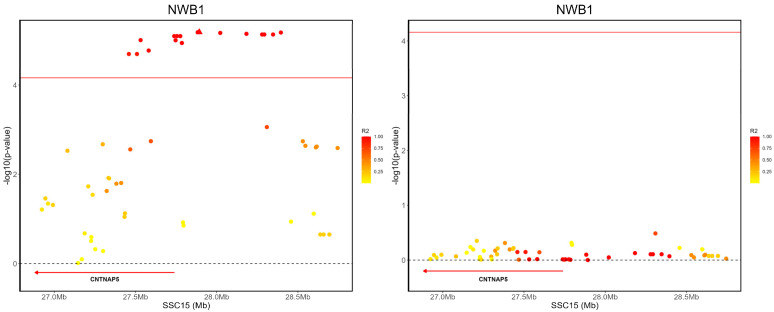
The regional association plot illustrates the primary signal (rs332278643) associated with NWB on SSC15 in the Large White population at the second parity. The plot displays negative log10 *p*-values of SNPs (*y*-axis) according to their chromosomal positions (*x*-axis). The red line indicates the genome-wide significance level. The primary SNPs are marked by red triangles. The left and right panels of the figure show the association results for NWB before and after conditional analysis on rs332278643, respectively. The color of the dots in the plot represents the level of linkage disequilibrium (LD) with the primary signal (rs332278643), where redder dots indicate higher LD levels, and yellower dots indicate lower LD levels.

A total of 17 SNPs located within the 27.45–28.39 Mb region on chromosome 15 were significantly associated with NWB traits in the first parity. We conducted a conditional analysis based on the top SNP rs332278643, complemented by LD analysis. It was found that these SNPs exhibit a high level of linkage with the top SNP rs332278643, with r2 values exceeding 0.8. Additionally, the *CNTNAP5* gene is located in proximity to this region (Figure 4). The *CNTNAP5* gene, disrupted by a paternal microdeletion at 2q14.3 in an autism spectrum disorder (ASD) study, is implicated in neural communication and connectivity, as evidenced by its association with autism spectrum disorders and additional rare missense mutations found in extended analyses [36]. This gene’s role in neurodevelopmental processes highlights its potential impact on cognitive functions and behavioral traits associated with ASD. Eight SNPs were significantly associated with NSB traits in the second parity; of these, four SNPs were located on chromosome 6, three on chromosome 11, and one on chromosome 14. The most significant SNP, rs342135123, was found on chromosome 6, near the GSE1 gene. The GSE1 gene was identified as a novel locus significantly associated with tardive dyskinesia in schizophrenia patients in a Genome-Wide Association Study [37]. Additionally, we conducted a conditional analysis using the prominent SNP rs342135123, further supported by LD analysis (Figure S7). In this analysis, we included the genotypes of the key pleiotropic SNP rs342135123 as a covariate in the mixed linear model (MLM), which resulted in the *p*-value for the association with NDF dropping below the anticipated threshold. This analysis confirmed that these SNPs are tightly linked to the primary SNP rs342135123, with r2 values exceeding 0.8. Individuals carrying the GG genotype at SNP rs342135123 showed an average NDF value that was 0.81 lower compared with those with the CC genotype and 0.50 lower compared with carriers of the GC genotype (Figure S8). Thus, this SNP also demonstrates significant breeding value.

One SNP rs81310054 was significantly associated with NDF traits in the first parity, with *PKD2* identified as the candidate gene. Eight SNPs located on chromosome 3 (from 60.15 Mb to 61.87 Mb) were significantly associated with NDF traits in the second parity, with SUCLG1 as the candidate gene. A conditional analysis was also conducted using the top SNP rs81304023, which was further supported by LD analysis (Figure S9). It was found that these SNPs are tightly linked to the primary SNP rs342135123, with r2 values exceeding 0.8. The *PKD2* gene encodes Polycystin-2 (PC2), a protein that functions as a Ca^2+^-permeable nonselective cation channel, playing a crucial role in Ca^2+^ signaling and cellular Ca^2+^ homeostasis. Mutations in PKD2 are responsible for Autosomal Dominant Polycystic Kidney Disease (ADPKD), highlighting its significant role in renal epithelial cell function and health [38]. The *SUCLG1* gene encodes succinate-CoA ligase, which plays a critical role in the mitochondrial energy metabolism pathway, and mutations in SUCLG1 are linked to significant clinical manifestations, including hepatopathy and hypertrophic cardiomyopathy [39]. One SNP rs321202796 was significantly associated with MUMM traits in the first parity, with *KIAA0930* identified as the candidate gene. The *KIAA0930* gene is implicated in influencing the cachexic phenotype in cancer cells, suggesting a role in cancer-associated wasting and metabolic dysregulation [40].

### 3.5. The Functional Enrichment Analysis for Litter Traits

The functional genes identified in this study are associated with the development of multiple tissues and the secretion of essential hormones during pregnancy, such as embryonic placenta development, peptidyl-arginine methylation, testis development, ovary development, sex hormone signaling, spermatogenesis, and oogenesis (Table S1). These factors are closely linked to the number of litters and the survival or mortality of piglets. Building on these results, future GWAS on swine reproductive traits should focus on these critical genetic markers for more precise breeding strategies. To effectively leverage these findings, it is advisable for breeding programs to consider incorporating these genetic markers to potentially enhance reproductive efficiency and outcomes, particularly in terms of litter size. This approach offers a practical framework that breeders could use to integrate genetic insights, thereby refining breeding strategies to improve reproductive traits.

## 4. Conclusions

In this study, we conducted a GWAS to identify SNPs and candidate genes associated with litter traits across the first two parities in the Large White pig population. We successfully detected 45 significantly related SNPs and 17 potential candidate genes, including *COG6*, *GSE1*, *CNTNAP5*, *IRF8*, *PAN3*, *SLC39A12*, *SUCLG1*, *DOCK9*, *GFRA2*, *OLFM3*, *PRMT6*, *WASF3*, *KIAA0930*, *MTHFSD*, *PKD2*, *TXLNB*, and *COL21A1*. These findings provide valuable tools for enhancing the quantity and quality of Large White litters through molecular marker-assisted or genomic selection. Additionally, this research deepens our understanding of the genetic basis of sow reproductive traits, facilitating targeted improvements and sustainable practices in swine breeding.

## Figures and Tables

**Table 1:** Summary statistics of litter traits in Large White populations.

Parity	Trait	N	Mean	SD	Minimum	Maximum	Heritability
1	TNB	2036	17.56	3.59	6	25	0.04
	NBA	1955	14.72	3.66	5	23	0.02
	NHB	1958	13.61	3.50	4	21	0.04
	rNHB	1978	77.27	16.60	15	100	0.02
	NWB	1991	0.89	1.34	0	5	0.01
	NDF	2022	0.02	0.15	0	1	0.01
	NSB	1937	1.88	1.66	0	6	0.01
	MUMM	1914	0.66	1.07	0	4	0.01
2	TNB	1332	18.53	3.34	6	25	0.04
	NBA	1306	15.59	3.64	5	23	0.06
	NHB	1306	14.14	3.27	4	21	0.05
	rNHB	1308	76.64	14.47	15	100	0.01
	NWB	1298	1.28	1.45	0	5	0.01
	NDF	1328	0.02	0.15	0	1	0.01
	NSB	1258	1.98	1.69	0	6	0.01
	MUMM	1292	0.61	1.02	0	4	0.01

TNB, Total number born; NBA, number of piglets born alive; NHB, number of healthy births; rNHB, the rate of NHB; NWB, number of weak births; NDF, number of deformed fetuses; NSB, number of stillborn; MUMM, mummified pig.

**Table 2:** Summary of GWAS results for first-litter size trait in the Large White pig population.

Trait	SSC	rs	Position	Allele	Maf	*p*	Candidate Gene
NHB	1	rs55618047	25284574	C/T	0.315315	6.05448 × 10^−5^	*TXLNB*
rNHB	10	rs81294332	44308657	A/G	0.180471	2.21049 × 10^−5^	*SLC39A12*
NWB	15	rs336401964	27459405	T/C	0.229584	2.02398 × 10^−5^	*CNTNAP5*
NWB	15	rs333569636	27509302	T/C	0.229439	2.02405 × 10^−5^	*CNTNAP5*
NWB	15	rs81304168	27531806	A/G	0.218686	9.90197 × 10^−6^	*CNTNAP5*
NWB	15	rs335973133	27581413	C/T	0.221593	1.69344 × 10^−5^	*CNTNAP5*
NWB	15	rs333665093	27738575	A/G	0.220866	8.04687 × 10^−6^	*CNTNAP5*
NWB	15	rs343368776	27746996	C/G	0.221011	9.97989 × 10^−6^	*CNTNAP5*
NWB	15	rs320619304	27755517	A/G	0.220866	8.04687 × 10^−6^	*CNTNAP5*
NWB	15	rs81305599	27775994	A/G	0.220866	8.04687 × 10^−6^	*CNTNAP5*
NWB	15	rs337432591	27785758	A/G	0.221593	1.14719 × 10^−5^	*CNTNAP5*
NWB	15	rs327976032	27883239	A/T	0.220575	6.59095 × 10^−6^	*CNTNAP5*
NWB	15	rs332278643	27892641	T/G	0.220575	6.57329 × 10^−6^	*CNTNAP5*
NWB	15	rs81478872	28021915	T/G	0.220285	6.82459 × 10^−6^	*CNTNAP5*
NWB	15	rs3470958220	28183106	T/C	0.219849	7.14878 × 10^−6^	*CNTNAP5*
NWB	15	rs327135140	28279474	G/T	0.219994	7.4039 × 10^−6^	*CNTNAP5*
NWB	15	rs81452141	28293171	A/C	0.219994	7.4039 × 10^−6^	*CNTNAP5*
NWB	15	rs322582049	28347013	A/G	0.219994	7.4039 × 10^−6^	*CNTNAP5*
NWB	15	rs338760245	28395070	T/C	0.219704	6.6822 × 10^−6^	*CNTNAP5*
NDF	8	rs81310054	131016420	A/C	0.104911	5.92047 × 10^−5^	*PKD2*
MUMM	5	rs321202796	4183089	T/C	0.121331	5.00831 × 10^−5^	*KIAA0930*

SSC, Sus scrofa chromosome; TNB, Total number born; NBA, number of piglets born alive; NHB, number of healthy births; rNHB, the rate of NHB; NWB, number of weak births; NDF, number of deformed fetuses; NSB, number of stillborn; MUMM, mummified pig.

**Table 3:** Summary of GWAS results for second-litter size trait in the Large White pig population.

Trait	SSC	rs	Position	Allele	MAF	*p*	Genes
TNB	4	rs344560170	113119104	C/T	0.387242	4.308 × 10^−5^	*PRMT6*
NBA	4	rs3474737609	116388595	A/T	0.0956117	4.258 × 10^−5^	*OLFM3*
NBA	11	rs341909772	14909631	T/C	0.447835	7.613 × 10^−6^	*COG6*
NBA	11	rs81431697	67830653	G/A	0.372857	3.524 × 10^−5^	*DOCK9*
NHB	7	rs341878379	29614695	A/C	0.406423	6.299 × 10^−5^	*COL21A1*
NHB	11	rs341909772	14909631	T/C	0.447835	1.759 × 10^−6^	*COG6*
NHB	15	rs1108768276	6547063	C/A	0.409183	6.827 × 10^−5^	*ENSSSCG00000053606*
NHB	15	rs320524315	6384510	C/T	0.343795	5.407 × 10^−5^	*ENSSSCG00000053606*
rNHB	6	rs320041489	2728087	T/C	0.402645	6.856 × 10^−5^	*MTHFSD*
NSB	6	rs333336780	2814834	C/T	0.274629	1.398 × 10^−5^	*IRF8*
NSB	6	rs334764615	2387727	T/C	0.321854	5.853 × 10^−5^	*MTHFSD*
NSB	6	rs336729684	2454772	T/C	0.273612	6.613 × 10^−5^	*MTHFSD*
NSB	6	rs342135123	3342934	G/C	0.380703	2.223 × 10^−6^	*GSE1*
NSB	11	rs329467410	5544200	C/T	0.106946	6.536 × 10^−5^	*PAN3*
NSB	11	rs345895836	5600114	C/T	0.0998256	1.604 × 10^−5^	*PAN3*
NSB	11	rs81430859	4296303	C/T	0.140221	4.744 × 10^−5^	*WASF3*
NSB	14	rs337919249	5225585	C/T	0.0893636	4.035 × 10^−5^	*GFRA2*
NDF	3	rs81304023	60157640	T/C	0.0838419	2.389 × 10^−5^	*SUCLG1*
NDF	3	rs81330647	60211109	T/C	0.0838419	2.389 × 10^−5^	*SUCLG1*
NDF	3	rs81327030	60216589	T/C	0.0838419	2.389 × 10^−5^	*SUCLG1*
NDF	3	rs332244679	60266762	G/A	0.0838419	2.389 × 10^−5^	*SUCLG1*
NDF	3	rs81299811	60914644	C/G	0.0841325	2.392 × 10^−5^	*SUCLG1*
NDF	3	rs336799801	61636339	C/T	0.0842778	2.743 × 10^−5^	*SUCLG1*
NDF	3	rs81244504	61851017	C/T	0.0844231	2.541 × 10^−5^	*SUCLG1*
NDF	3	rs81326050	61878711	A/G	0.0839872	2.391 × 10^−5^	*SUCLG1*

SSC, Sus scrofa chromosome; TNB, Total number born; NBA, number of piglets born alive; NHB, number of healthy births; rNHB, the rate of NHB; NWB, number of weak births; NDF, number of deformed fetuses; NSB, number of stillborn; MUMM, mummified pig.

## Data Availability

The data presented in this study are available on request from the corresponding author due to the ongoing nature of the research project, which includes other unpublished work. This restriction is in place to protect the integrity and confidentiality of the ongoing research.

## Supplementary Materials

The following supporting information can be downloaded at https://www.mdpi.com/article/10.3390/ani14192874/s1, Figure S1: PCA Plots for Large White Pig Population. Left: 2D PCA using PC1 and PC2. Right: 3D PCA using PC1, PC2, and PC3 to explore genetic diversity; Figure S2: LD Decay Plot for Large White Pig Population; Figure S3: Q-Q plots of GWAS with SNPs for litter traits at the first parity in the Large White population; Figure S4: Q-Q plots of GWAS with SNPs for litter traits at the second parity in the Large White population; Figure S5: The genotype effect plot of the SNP rs341909772, which is associated with NBA in the Large White population at the second parity, is displayed. (** *p* < 0.001); Figure S6: The genotype effect plot of the SNP rs81294332, which is associated with rNHB in the Large White population at the first parity, is displayed. (** *p* < 0.001); Figure S7: The regional association plot illustrates the primary signal (rs333336780) associated with NSB on SSC6 in the Large White population at the second parity; Figure S8: The genotype effect plot of the SNP rs342135123, which is associated with NSB in the Large White population at the second parity, is displayed. (** *p* < 0.001); Figure S9: The regional association plot illustrates the primary signal (rs81304023) associated with NDF on SSC3 in the Large White population at the second parity; Table S1: Significant Gene Ontology and KEGG Pathway Analyses for Eight Reproductive Traits.
