# Supplementary figures and images for: Genome-Wide Association Study of Reproductive Traits in Large White Pigs
Source: Animals (Basel). 2024 Oct 6;14(19):2874. doi: 10.3390/ani14192874 (PMC11475698; doi:10.3390/ani14192874)

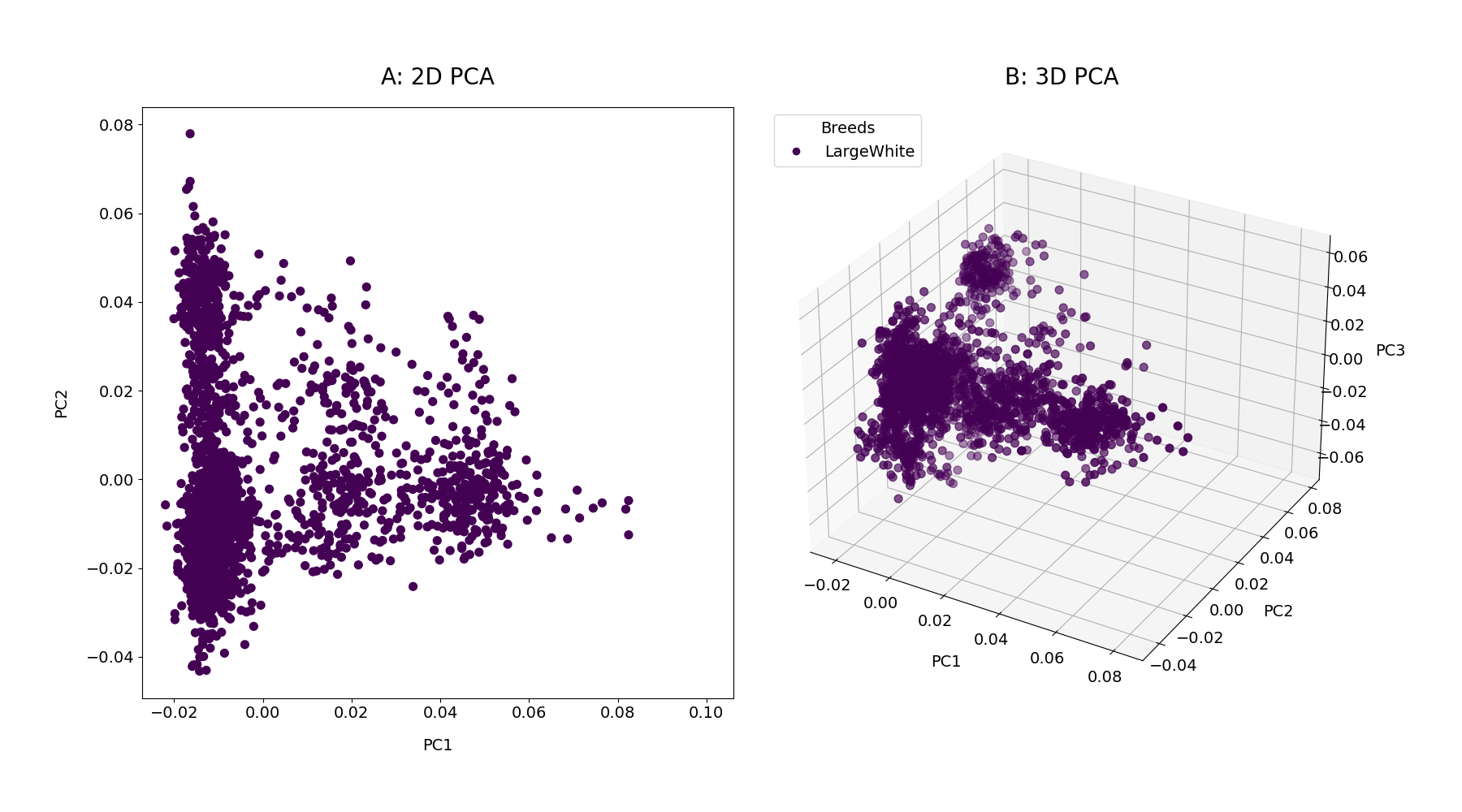

Supplement: Supplementary file 1 [file animals-14-02874-s001.zip › Figure S1.png]

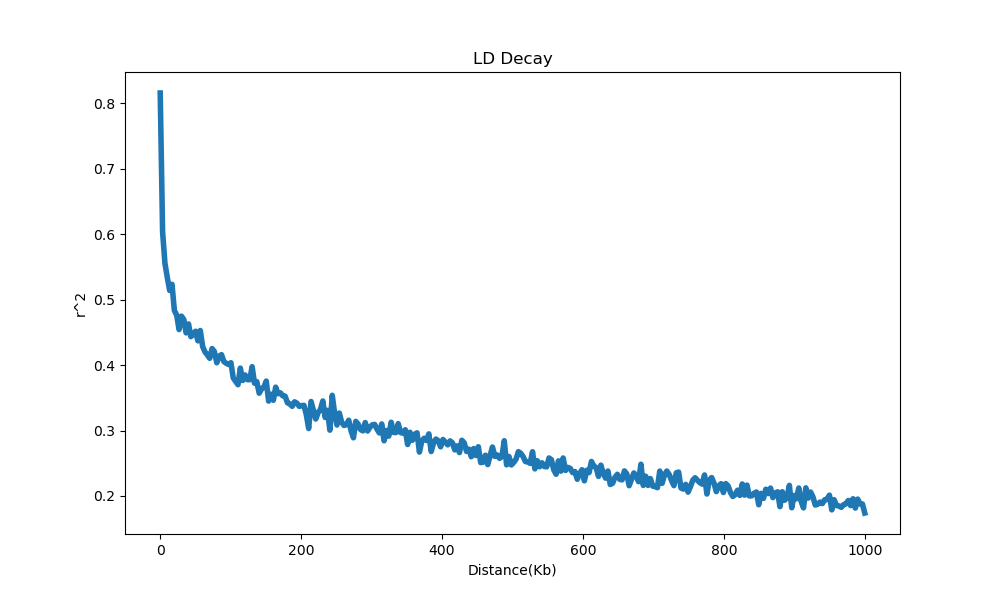

Supplement: Supplementary file 1 [file animals-14-02874-s001.zip › Figure S2.png]

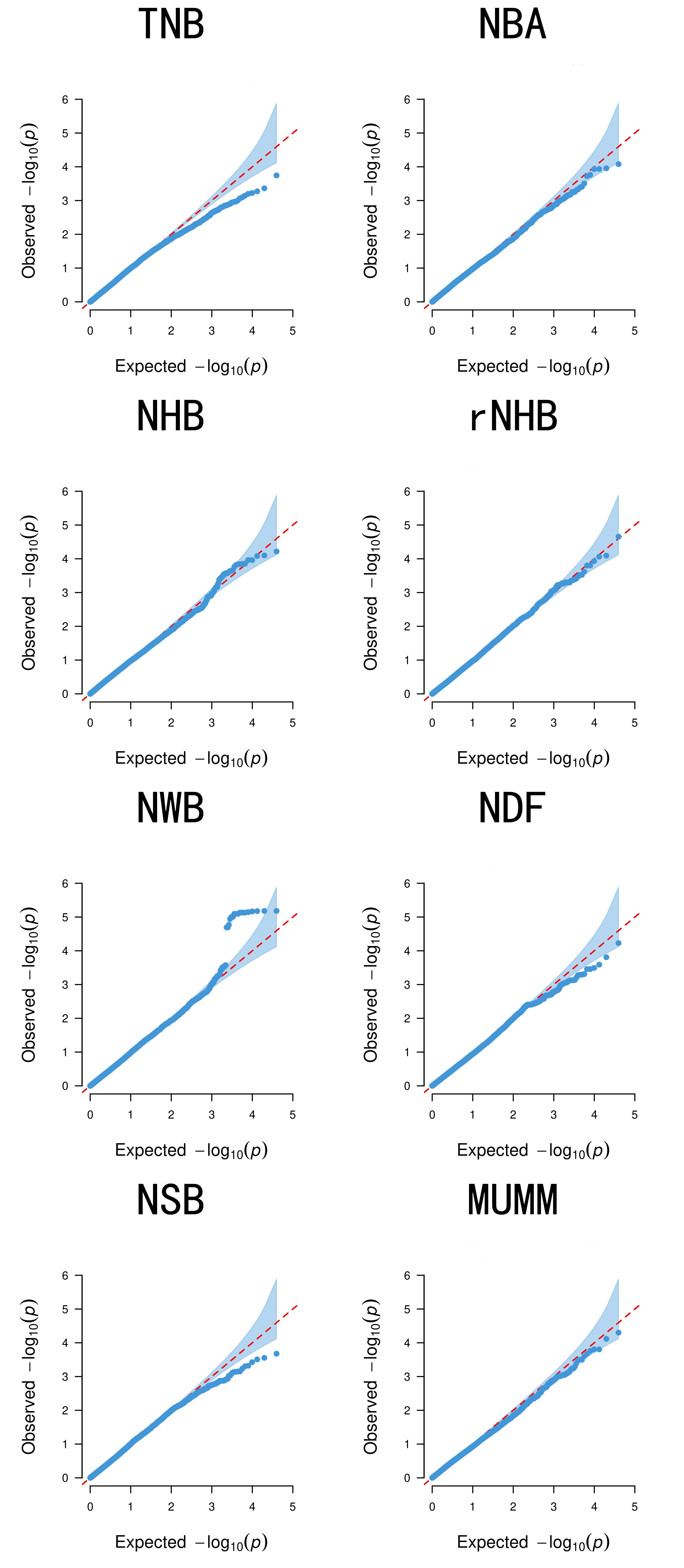

Supplement: Supplementary file 1 [file animals-14-02874-s001.zip › Figure S3.jpg]

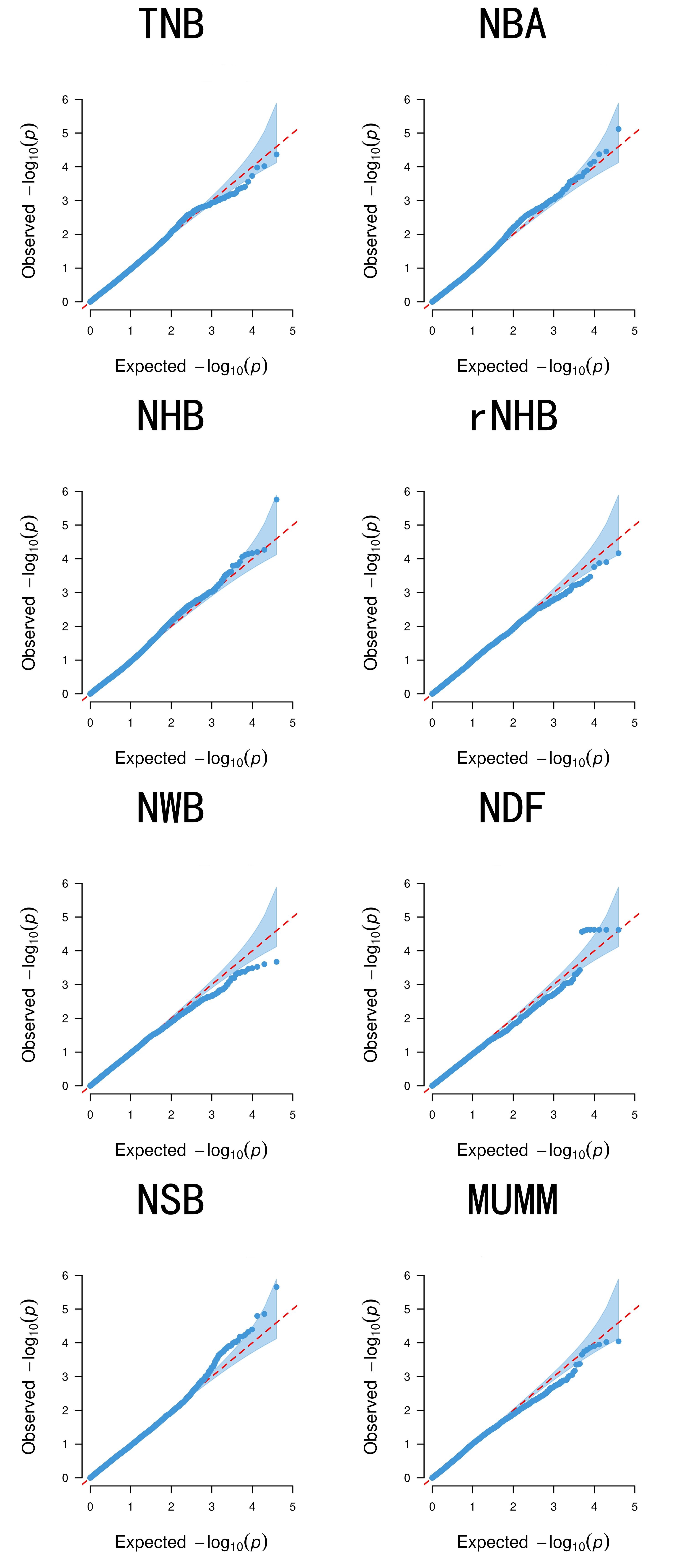

Supplement: Supplementary file 1 [file animals-14-02874-s001.zip › Figure S4.jpg]

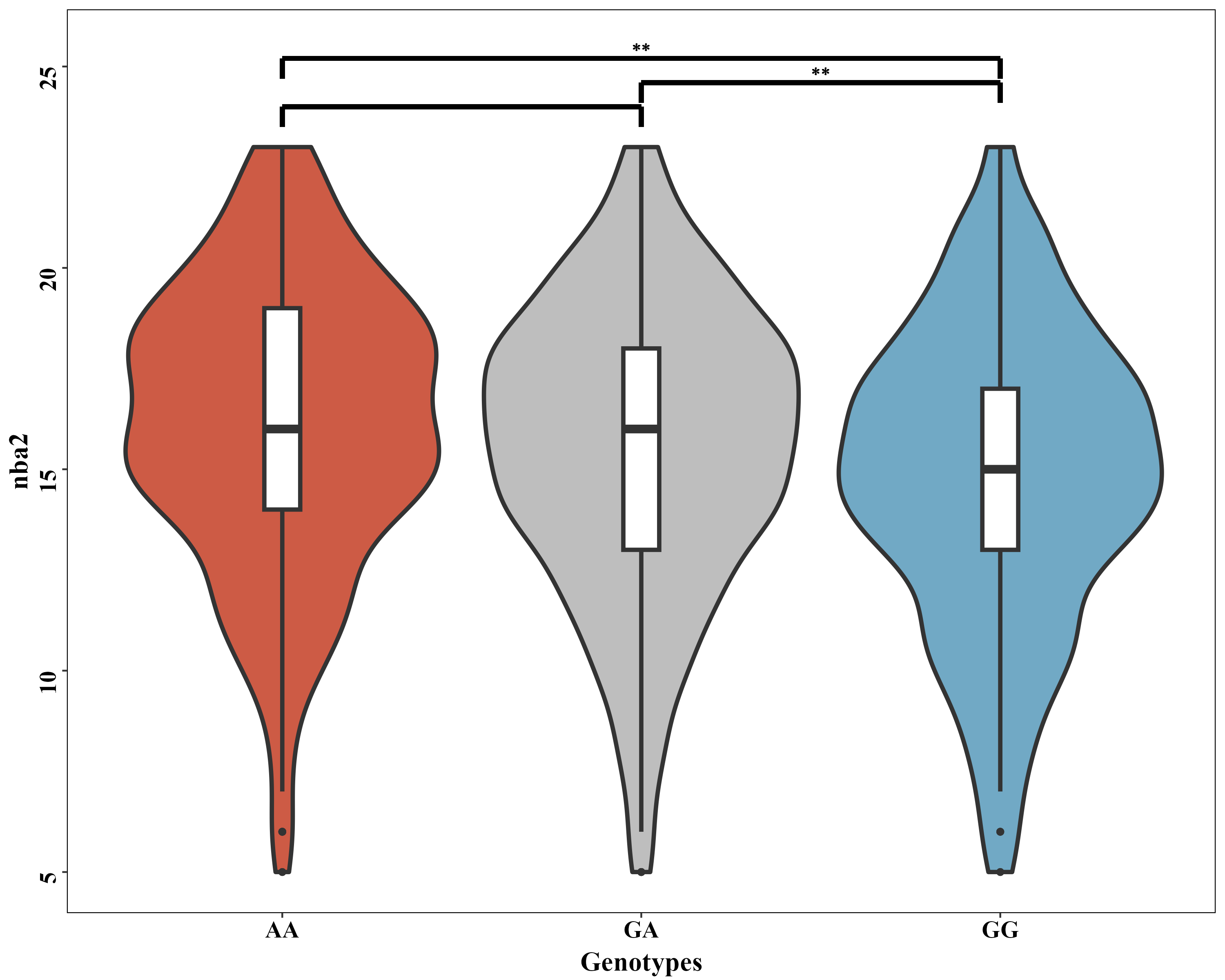

Supplement: Supplementary file 1 [file animals-14-02874-s001.zip › Figure S5.png]

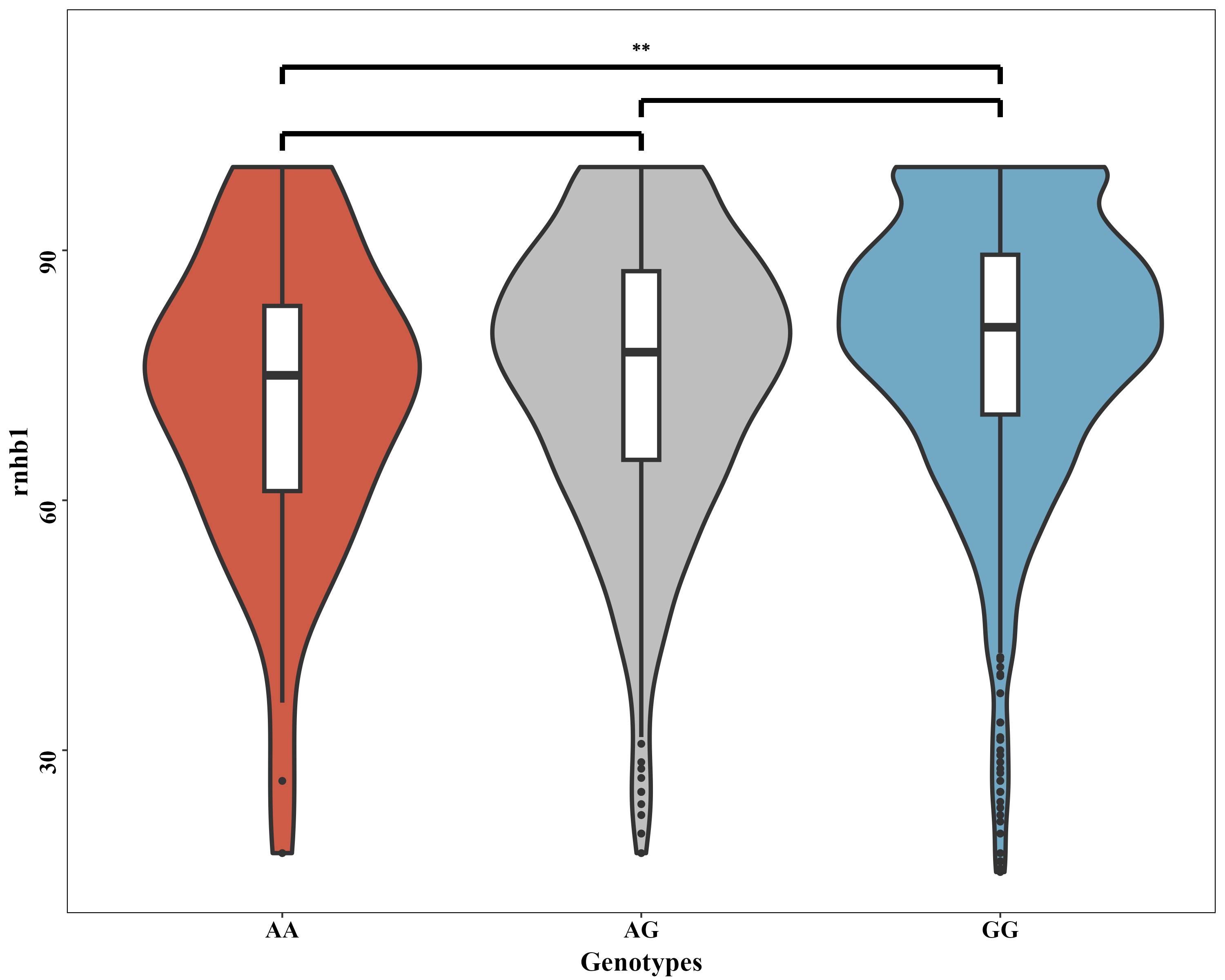

Supplement: Supplementary file 1 [file animals-14-02874-s001.zip › Figure S6.png]

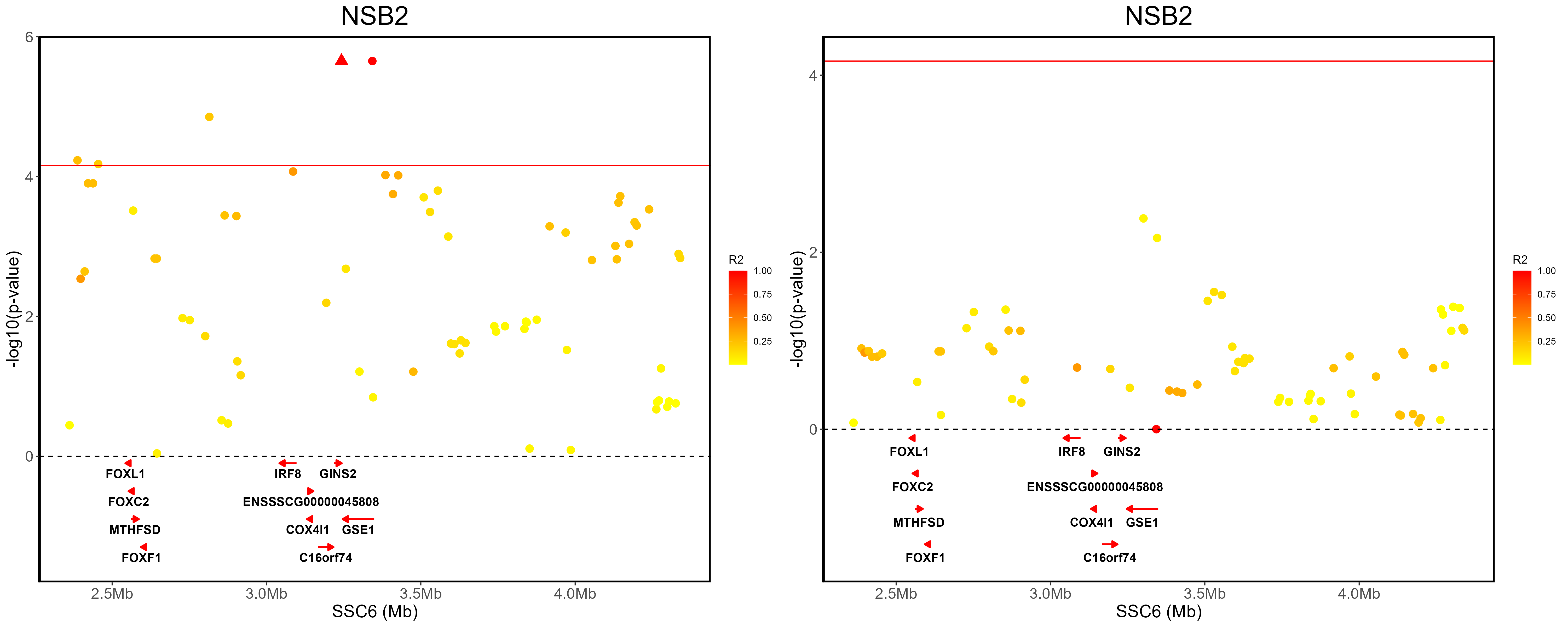

Supplement: Supplementary file 1 [file animals-14-02874-s001.zip › Figure S7.png]

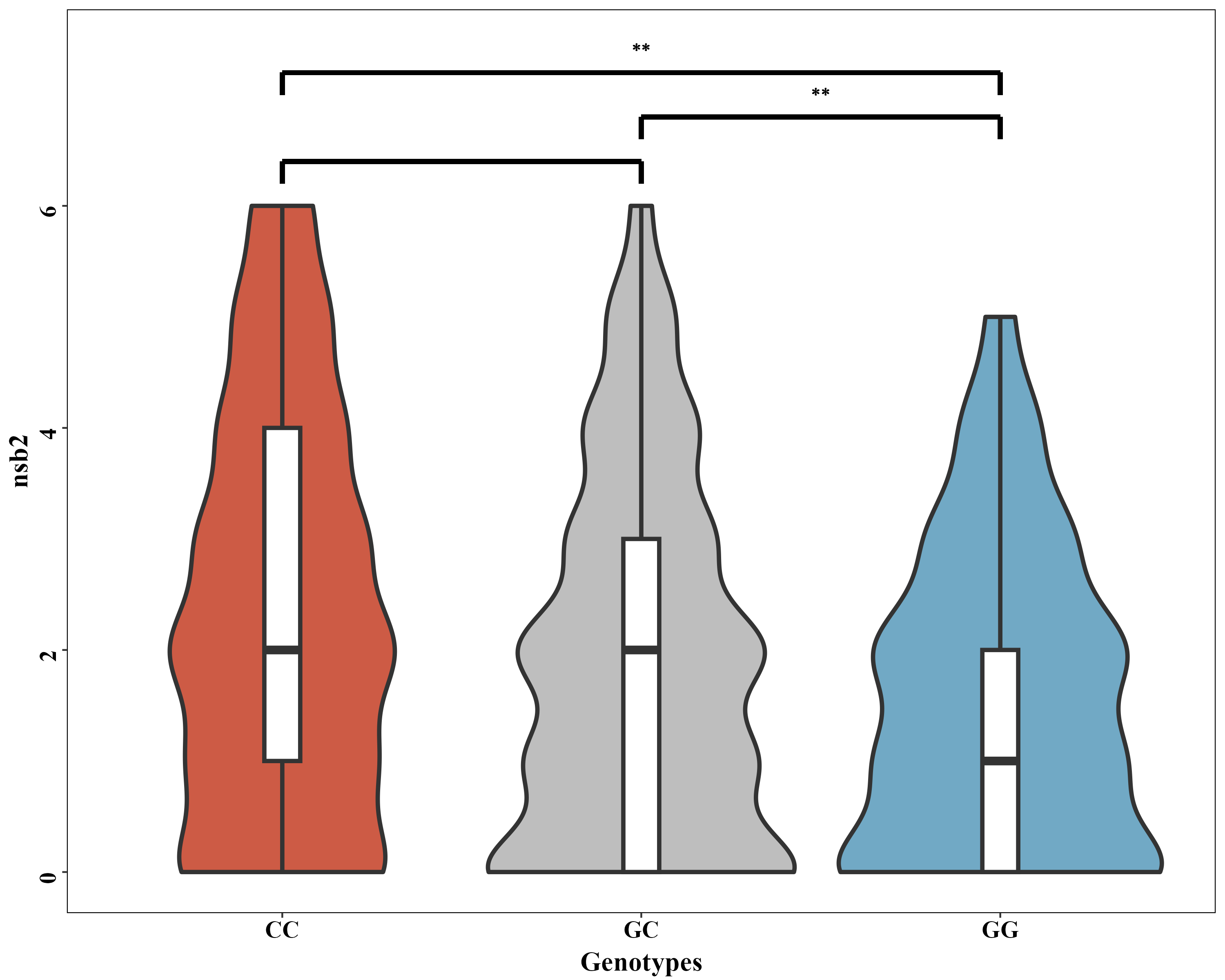

Supplement: Supplementary file 1 [file animals-14-02874-s001.zip › Figure S8.png]

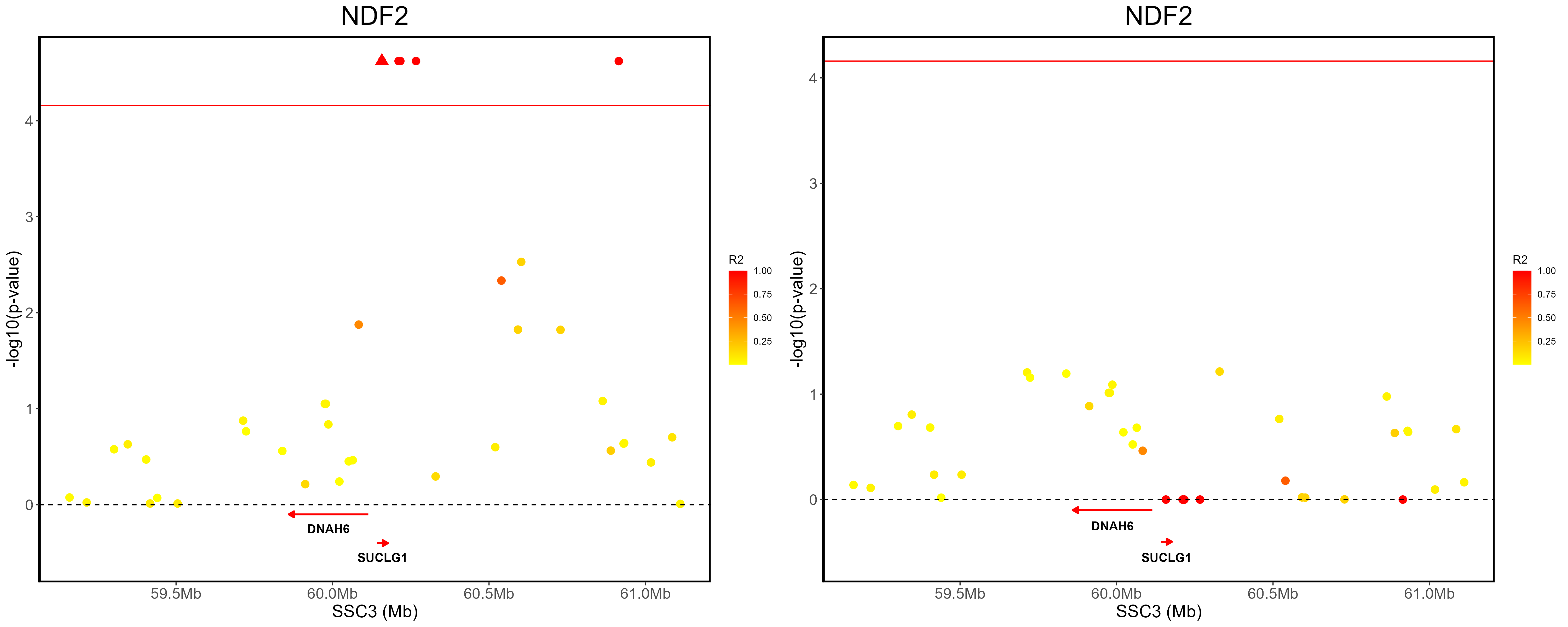

Supplement: Supplementary file 1 [file animals-14-02874-s001.zip › Figure S9.png]
